# Localization of epileptic foci in Children with childhood absence epilepsy by magnetoencephalography combined with synthetic aperture magnetometry^☆^

**DOI:** 10.1016/S1674-8301(11)60035-3

**Published:** 2011-07

**Authors:** Xiuxiu Hu, Jingde Dong, Xiaoshan Wang, Ting Wu, Lu Yang, Xiaopeng Lu

**Affiliations:** a Department of Geriatric Neurology, Nangjing Brain Hospital, Nanjing, Jiangsu 210029, China;; b Magnetoencephalography Center, Nangjing Brain Hospital, Nanjing, Jiangsu 210029, China;; c Department of Neurology, Nanjing Children's Hospital, Nanjing, Jiangsu 210029, China.

**Keywords:** childhood absence epilepsy, magnetoencephalography, synthetic aperture magnetometry, epileptic foci

## Abstract

This present study was aimed to investigate the localizable diagnostic value of magnetoencephalography (MEG) combined with synthetic aperture magnetometry (SAM) in childhood absence epilepsy (CAE). Thirteen CAE patients underwent MEG detection at resting state and after hyperventilation, and then the epileptic foci were located by SAM. In the thirteen CAE patients, epileptic foci were found in five cases (38.5%), and they were all located in the bilateral frontal lobe, suggesting that the frontal lobe in some CAE patients may serve as the epileptic foci. Our findings indicate that MEG combined with SAM could be of diagnostic value in localizing the epileptic foci in certain CAE patients.

## INTRODUCTION

Childhood absence epilepsy (CAE) is a common form of idiopathic generalized epilepsy (IGE), accounting for approximately 5%-15% of all childhood epilepsies[1]. With an onset age between 3 to 12 years (peak manifestation age 6 to 8 years old), the incidence of CAE is higher among girls. Typical CAE is characterized by sudden, frequent, and brief losses of awareness with or without motor automatisms. Pauses of ongoing activities and speech, blackout, and face slack are reported, but patients do not fall. Absence seizures occur with a frequency of a few times to hundreds of times per day with sudden occurrence and relief. The seizure can last for 3 s to as long as 40 s. Absences occur spontaneously but are also readily provoked by hyperventilation and occasionally by photic stimulation. The electroencephalography (EEG) reveals bilateral, synchronous symmetrical 3 Hz spike-and-slow-wave-discharge (SWD) on a normal background activity[2]–[4].

Magnetoencephalography (MEG) is a functional cerebral imaging technique that non-invasively records extracranial magnetic fields generated by the electrical activity of the brain[5]. It is widely used in nervous system diseases and clinical researches. MEG is conventionally required for preoperative identification of epileptogenic and eloquent cortical regions before epilepsy surgery. Magnetoencephalographic spike sources can be used to localize the epileptogenic zone. Synthetic-aperture magnetometry (SAM) is a method for analysis of data obtained from MEG and EEG. SAM is a nonlinear beam forming approach, which can be thought of as a spatial filter. SAM is a very useful method for source current imaging. SAM with excess kurtosis (g2) analysis was performed for localization of the epileptogenic focus[6]–[7]. It can significantly reduce processing time and improve the rate of clinical diagnosis.

Current absence epilepsy studies focus on aspects of animal models, genetics or EEG. However, MEG detection combined with SAM analysis in localization diagnosis of CAE has not been previously reported. Therefore, this study focused on MEG detection combined with SAM analysis of children with absence epilepsy in locating epileptic foci in order to investigate the localizable diagnostic value of MEG combined with SAM in CAE.

## MATERIALS AND METHODS

### Subjects

Thirteen subjects were recruited from the Department of Neurology of Nanjing Children's Hospital from October 2008 to June 2009. Everyone of them met the CAE diagnostic criteria of the International League Against Epilepsy (ILAE) in 2001[8] and received a confirmed diagnosis of typical CAE. The inclusion criteria were as follows: 1) typical absence seizures appeared as the initial seizure type at 3-12 years of age; 2) very frequent absence seizures occurred multiple times per day; 3) absence seizures were associated with bilateral, symmetric, and synchronous releases of regular 3 Hz SWDs in an EEG with normal background; 4) general physical and neurological examinations returned normal results; 5) neuroradiological examinations (CT or MRI) were normal. The exclusion criteria were as follows: 1) the presence of an implant of metal such as cochlear implant devices, a pacemaker or having other metals that can produce visible magnetic noise in the MEG data; 2) history of major psychiatric disease; 3) history of autism or pervasive development disorder; 4) being unable to keep head still for approximately 20 min; 5) clinically significant systemic organic disease; 6) the subjects or parents/legal guardians were potentially unable to be compliant with or complete the study. This study was approved by Ethics committee of Nanjing Brain Hospital and Nanjing Children's Hospital, and all guardians of subjects signed the informed consent.

### Methods

All children were required to stop taking anti-epileptic drugs two days before their MEG detection as well as staying up late and getting up early so as to reduce sleeping time. During the examination, to obtain children' cooperation, all subjects were well informed to act in the following ways during the detection: keeping quiet, being physically relaxed, staying still especially with the head, trying to avoid teeth clenching, frowning, swallowing and other facial movements except for hyperventilation movements during hyperventilation trials, keeping eyes slightly closed, and avoiding blinking and eye movement.

### MEG recording and analysis

CTF-275 guided by the whole head type magnetoencephalography system (VSM Medical Technology Company, Canada) was employed for MEG detection. A whole head 275-SQUID sensors MEG device simultaneously measured the corresponding induced magnetic fields. The head position was determined with localization coils at the nasion and preauricular fiducial points. Before and after each MEG recording, these points were localized by detecting the magnetic signals transmitted by the three coils. The two localizations were compared to check the head movement of each participant during the experimental session. In the direction perpendicular to the scalp, the brain magnetic field was measured and the magnetic source signal was collected. The information was stored in the computer. The coordinate system was set in each child using the digitizer. The rightward direction through the points before the two ears was set as positive in X-axis, and the forward direction from the bridge to tip of the nose was set as positive in Y-axis, and Z-axis was perpendicular to the X- and Y-axis, with the upward direction being its positive direction. While MEG signal was acquired, data were digitally filtered at a bandpass width 0.03-3.00 Hz in off-line data analysis and digitized at a sampling rate of 1,200 Hz. For the signal acquisition process, spontaneous, cerebral activities were recorded at an eye-closed resting state for approximately 2 min, for a repetitive 10 times, followed by a 3-min hyperventilation trial, and a 2-min recording.

### MRI acquisition

Anatomical images were obtained during a separate session with a 1.5-T Sigma magnetic resonance imaging (MRI) scanner for each patient. All patients underwent cranial MRI positioning: Head position was controlled using three coils placed at the left and right preauricular points and the nasion, serving as reference landmarks for anatomical MRI registration. In order to co-register the MRI and MEG data, three fiducial markers were placed on the MRI at the same locations used during MEG data acquisition. With sponge pad to get the head fixed, superconducting MRI instrument (NV/I 1.5T, Signa, GE, USA) was applied and quadrature head coil was adopted. T1-SPGR axial images were acquired through line 3D-SPGR sequential scanning. Scan parameters were as follows: field of view (FOV) = 24×24×16.2, matrix = 256×256×54, repetition time (TR) = 33.333 ms, echo time (TE) = 9 ms, inversion time = 0, number of averages = 1, imaging frequency = 63.849,208.

### Magnetic source imaging analysis and postprocessing

The scalp data used for source reconstruction were the raw MEG data of reference and seizure periods. Raw MEG data was transferred to the MEG workstations. Software designed by Canadian CTF Company was used to superpose MEG physiological data on MRI anatomical information to produce magnetic source imaging (MSI). MSI was defined as the integration of functional data derived from MEG recordings with structural MRI. MSI showed functional and structural information in a single image. Magnetic source signals within the frequency range of 20-70 Hz were selected and automatically calculated by SAM analysis. Head model standardizing was then applied to make sure that the processed signals were compatible with each participant's MR surface. As a result, the magnetic signal source was displayed in the corresponding parts of MRI images so as to form the ultimate brain function image.

## RESULTS

### Patient demographic and disease characteristics

A total of 13 subjects met our inclusion criteria and were recruited for the current study. They included 3 males and 10 females. The subjects aged 3 years 2 months to 12 years with an average of (8.4±3.2) years. Their course of disease was 3 months to 3 years, (1.6±0.9) years on average. Seizure onset ranged from 5 to 20 times per day. The seizure duration ranged from 6-30 s. All the diagnosed patients post-release accepted the medical treatment of sodium valproate tablets or Depakine syrup. The seizure frequency was significantly reduced, and the remaining seizures in children were generally 2 to 3 times/d, or every 2 to 3 d for 1 up to 10 attacks per day with each episode lasting 6-10 s. One of the cases was nearly 1 y with no further episodes before MEG detection.

MEG and MRI information of all thirteen subjects was successfully obtained. However, the hyperventilation operation of one subject was not standardized. Thus, we did not capture the magnetic source signal of hyperventilation, which though did not affect the results. According to the SAM analysis, epileptic foci located in the bilateral frontal lobe were found in five patients, while no clear localization of epileptic foci was detected for the remaining eight children. The positive rate of MEG detection combined with SAM analysis in locating the epileptic foci of CAE was 38.5%. The five patients (two boys and three girls) aged between 6 years 8 months to 12 years with an average age of (8.4±3.2) years. Their course of disease ranged from 6 months to 20 months with an average of 14 months. Still having relatively high seizure frequency, most of them failed to be treated effectively. Three of them had an onset of 6 to 10 times per day. The other two had 3 to 5 times per day and no further attack for one year, respectively.

**Fig. 1:**
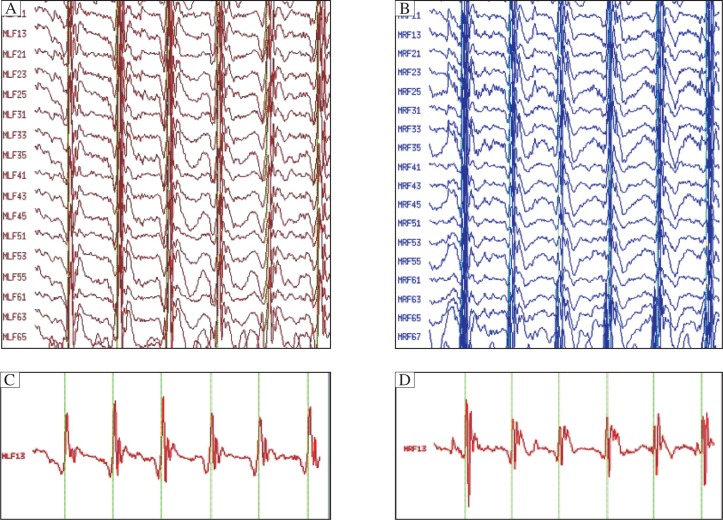
MEG magnetic source signal waveform of children with possible epileptic foci in bilateral frontal lobe. Overall magnetic source signal waveforms are shown in the left (A) and right (B) frontal lobe. Single magnetic source signal waveforms are shown in the left (C) and right (D) frontal lobe.

**Fig. 2:**
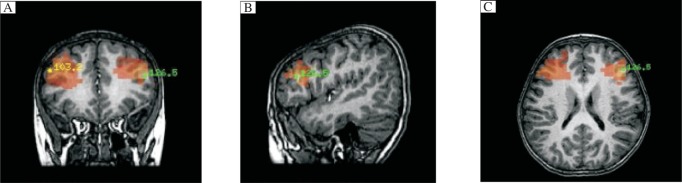
MEG brain function image map of children with possible epileptic foci in the bilateral frontal lobe. Red area refers to the possible epileptic focus. A: coronal position, B: sagittal position, C: horizontal position.

**Fig. 3:**
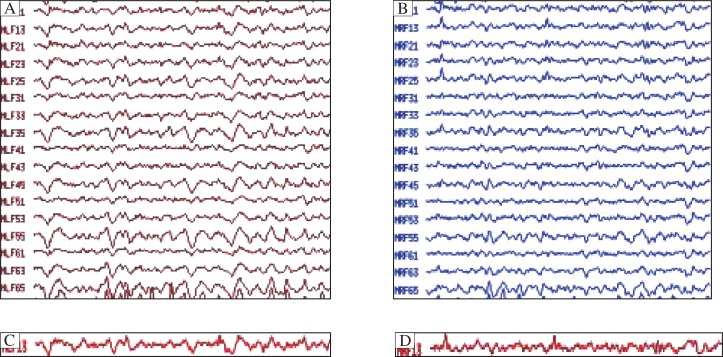
MEG magnetic source signal waveform of children with unclear epileptic foci in the frontal lobe. Overall magnetic source signal waveforms are shown in the left (A) and right (B) frontal lobe. Single magnetic source signal waveforms are shown in the left (C) and right (D) frontal lobe.

**Fig. 4:**
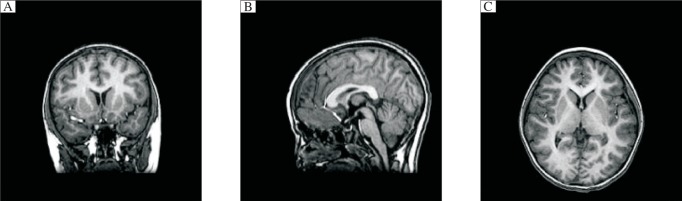
MEG brain function image map of children with unclear epileptic foci in the frontal lobe. A: coronal position. B: sagittal position. C: horizontal position

According MEG detection combined with SAM analysis, the magnetic source waveform and possible epileptic foci location magnetic source imaging map at rest and after hyperventilation of the children with absence epilepsy are shown in ***Fig. 1*** (MEG brain function image map of children with possible epileptic foci in the bilateral frontal lobe) and ***Fig. 2*** (MEG brain function image map of children with possible epileptic foci in the bilateral frontal lobe), and the magnetic source waveform and magnetic source imaging map of children with unclear epileptic foci locations are shown in ***Fig.3*** (MEG brain function image map of children with unclear epileptic foci in the bilateral frontal lobe) and ***Fig.4*** (MEG brain function image map of children with possible epileptic foci in the bilateral frontal lobe).

## DISCUSSION

MEG performs noninvasive functional imaging by recording the magnetic flux on the head surface associated with electrical currents in activated sets of neurons. MEG has rapidly evolved in the last two decades because of the introduction of whole head systems and advances in computer technology. MEG is now the imaging modality of choice where a precise and high degree of localization is required. MEG can be used to localize the primary sensory cortices (visual, auditory, or somatosensory), areas involved in receptive language function, and the irritative zone in epilepsy patients, and identify children with anomalous language development[9],[10]. In comparison to EEG, MEG has a much better spatial resolution[11]. In comparison to other neuroimaging modalities such as MRI, MEG has a very high temporal resolution[11]. Routinely, MEG can attain a temporal resolution of less than a millisecond and, under optimal circumstances, spatial resolution of several millimeters[12]. MSI is a combination of MEG and coregistered MRI that is increasingly being used in the non-invasive prooperative evaluation of patients with refractory partial epilepsy to localize the magnetic correlate of interictal epileptiform discharges[9]. Especially in the preoperative evaluation of patients with refractory partial epilepsy, surgical guidance and other aspects of program development play an increasingly important role[11],[13]–[15]. Dynamic MSI is a technology developed by us to render a set of sequential MSI to reveal dynamic changes of brain activation in spatial, temporal and frequency domains.

Recently developed SAM is a new MEG signal analysis introducing a high performance spatial filtering technique. SAM is not an inverse solution like dipole analysis, but rather an adaptive beamformer for estimating source activity at each selected voxel inside of the brain. SAM can estimate source changes as a function of time, or power changes subjected to statistical analysis from the non-averaged raw MEG data[16]. Spatial filtering can be applied to MEG data by means of SAM, and SAM virtual sensor analysis can be used to estimate the strength and temporal course of the epileptic source in the region of interest[17]. The SAM virtual sensor analysis revealed morphological characteristics, location, and distribution of epileptiform discharges similar to those shown by subdural EEG recordings. By using SAM it is possible to predict intracerebral interictal epileptiform discharges in the region of interest from noninvasively collected preoperative MEG data[17]. SAM can choose different weighting factors automatically according to the distance of each sensor from the brain signal source, which increases the signal to noise ratio of MEG signals substantially. SAM has been widely used in preoperative epileptic focus localization.

So far, possible mechanisms for childhood absence epilepsy have not been fully clarified. There were several main theories[18]: such as the “centrencephalic” theory proposed in 1954, the “thalamic clock” theory proposed in 1991, “corticoreticular” theory postulated in 1968, and spike-wave discharges are linked to the thalamocortical mechanisms that generate spindles. Rhythmic spindle oscillations generated in the thalamus are transformed into spike-wave discharges when the cortex is hyperexcitable. A study in 2002 confirmed in epileptic rats that a functionally intact thalamocortical network is required for the generation of spike-wave discharges[19]. The corticothalamic interrelationships were investigated by means of nonlinear association signal analyses of multiple spike-wave discharges. This showed a consistent focus within the perioral region of the somatosensory cortex. From this focus, seizure activity generalizes rapidly over the cortex. During the first cycles of the seizure the cortex drives the thalamus, while thereafter the cortex and thalamus drive each other, thus amplifying and maintaining the rhythmic discharge. In this way the “cortical focus” theory for generalized absence epilepsy bridges cortical and thalamic theories. The thalamus, especially the reticular nucleus, plays a major role, as does the frontal cortex, mainly the dorsolateral and orbital frontal areas, to the extent that some investigators have concluded that absence seizures are not truly generalized, but rather have selective cortical networks, mainly ventromesial frontal areas and the somatosensory cortex. The latter network is a departure from the more popular concept of a generalized epilepsy. Between the “centrencephalic” and “corticoreticular” theories, a “unified” theory is presented[20],[21].

Roche-Labarba *et al.*[22] used near infrared spectroscopy (NIRS) technology to study 6 children with absence epilepsy. The results showed that generalized SWDs were associated in the frontal area with an oxygenation (beginning 10 s before the SWD) followed by rapid deoxygenation. The oxygenation with [HbT] increased before returning to the baseline. This study emphasizes the role of the frontal lobe in generating SWD. Holmes *et al*.[23] found that the onset of seizures was typically associated with the activation of discrete, often unilateral areas of the dorsolateral frontal or orbital frontal lobe. Consistently across all seizures, the negative slow wave was maximal over the frontal cortex, and the spike that appeared to follow the slow wave was highly localized over the frontopolar regions of the orbital frontal lobe. In addition, sources in the dorsomedial frontal cortex were engaged for each spike-wave cycle. Tucker *et al*.[24] applied advanced methods of electrical source analysis to dense array (256-channel) electroencephalographic recordings of spike-wave discharges of absence spells. Neither the onset nor the spread of these seizures is generalized. Rather, the slow waves of the discharges are restricted to the frontotemporal networks, and the spikes represent a highly localized and stereotyped progression of electrophysiological activity in the ventromedial frontal networks. This specificity of the frontal cortical discharges suggests the hypothesis that absence spells are associated with pathology in a circuit comprised of the ventromedial frontal cortex, rostral thalamic reticular nucleus, and limbic nuclei of the thalamus. Disrupted in absence, this circuit appears to regulate important aspects of the voluntary control of conscious attention. Clemens *et al*.[25] believe that the prefrontal area of increased activity corresponds to the area that is essential in the buildup of the ictal spike-wave paroxysms (absence seizures). The frontal area of decreased activity might be related to the cognitive deficit described in IGE patients. Recently, Sakurai *et al*.[26] demonstrated the contribution of default mode network that exists in the medial aspect of the frontal lobe, in the genesis of juvenile absence epilepsy.

This study combined MEG detection with SAM analysis identified some part of the brain in children with absence epilepsy may be the epileptogenic zone; the epileptic foci were all found in the frontal, further indicating that the frontal lobe plays an important role in absence seizures. Our results were consistent with the “focal cortical theory” of absence epilepsy as well as findings of EEG researches on childhood absence epilepsy, which showed bilaterality.

The present study indicated that the seizure frequency of patients whose epileptic foci were found by MEG detection was higher than those who tested negative, indicating that the higher the frequency of attacks was, the more positive rate of epileptogenic zone was. Many researches emphasize the important role that the hypothalamus plays in absence epilepsy[27]–[30]. However, epileptic foci were only found in the frontal lobe in this study. This finding does not rule out that the epileptogenic zone may exist in other parts of the brain. Possible reasons could be: 1) With the extension of the distance, the magnetic field deamplification is more obvious. The farther of the distance is, the worse the magnetic field signals get. MEG is less sensitive to epileptic foci in deep brain regions. That may explain why this study failed to find epileptogenic zone in the hypothalamus. 2) As the children of absence epilepsy have received drug treatment after diagnosis and most of the treatment effects were effective, effective control of absence seizures may have some influence on MEG clinical studies. From this perspective, the treatment and disease severity of absence epilepsy can be judged by MEG. 3) Our results cannot cover all of the potential epileptogenic zones due to small sample size, which needs to be further expanded.

In summary, with MEG detection combined with SAM analysis, this study found that children with absence epilepsy may have epileptogenic foci, part of which in the bilateral frontal lobe. The frontal lobe plays an important role in the occurrence and development of CAE. MEG detection combined with SAM could be of a certain diagnostic value in localizing the epileptogenic foci of CAE; however, our study could not identify the origin of epileptic foci of childhood absence epilepsy. Further dynamic study is needed to ascertain the problem. This study also provided an objective basis for the assessment of severity and the effects of drug treatment of CAE. We believe that along with improvement in MEG detection technology and with advancement in analyzing software, researches can go beyond the preliminary epileptogenic foci localization and the pathophysiological mechanisms of childhood absence epilepsy, such as the origin of epileptic seizure foci, and localization and propagation path will be better understood.
